# Supplementary figures and images for: Bacterial virulence plays a crucial role in MRSA sepsis
Source: PLoS Pathog. 2021 Feb 25;17(2):e1009369. doi: 10.1371/journal.ppat.1009369 (PMC7942999; doi:10.1371/journal.ppat.1009369)

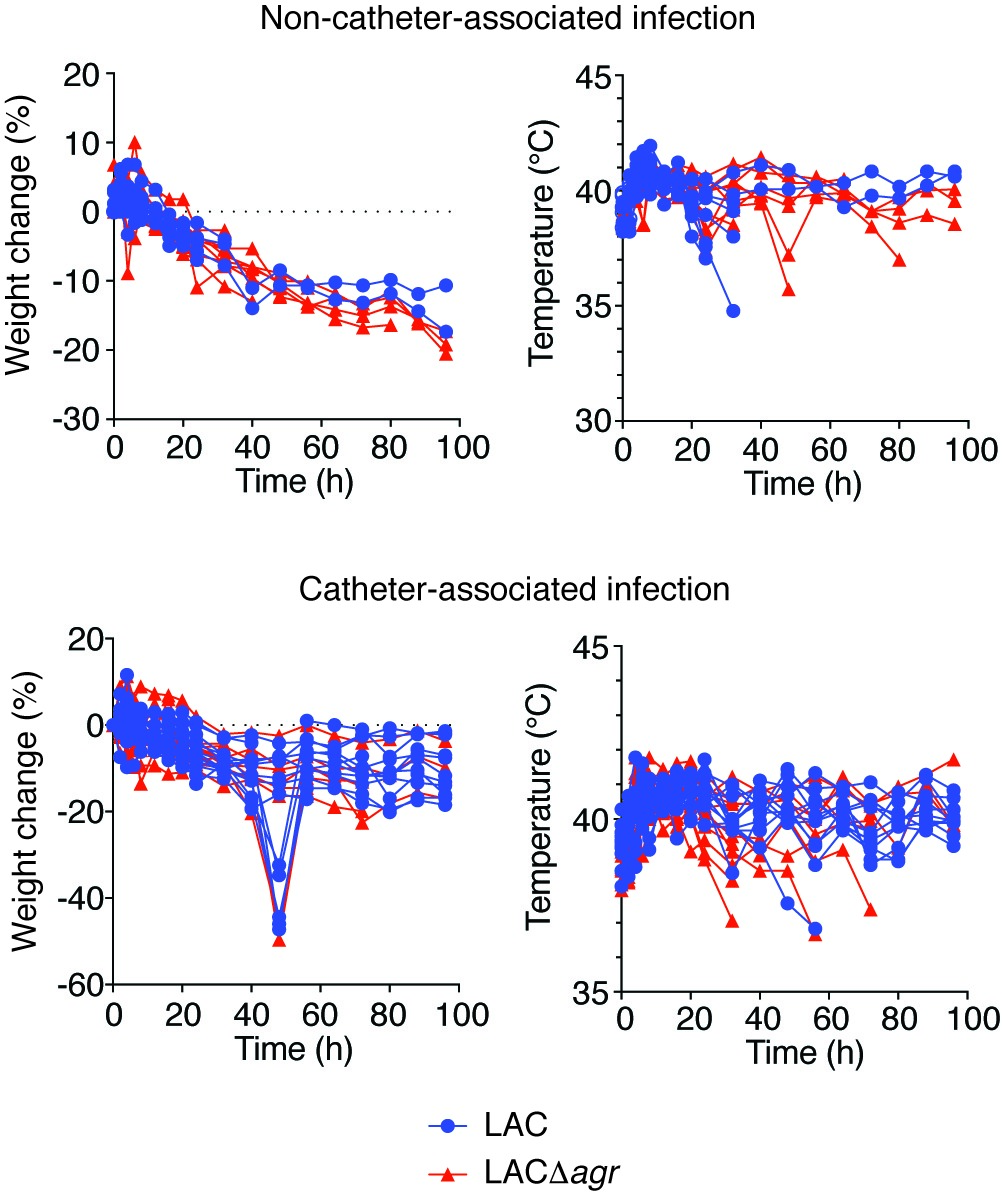

Supplement: S1 Fig — Weight change and rectal temperature over time in the non-catheter-associated and the catheter-associated sepsis models. The transient dip at ~ 48 h in weight for some animals in the catheter-associated model was due to those rabbits not eating. After critical care (forced feeding) was activated, they recovered. (TIF) [file ppat.1009369.s001.tif]

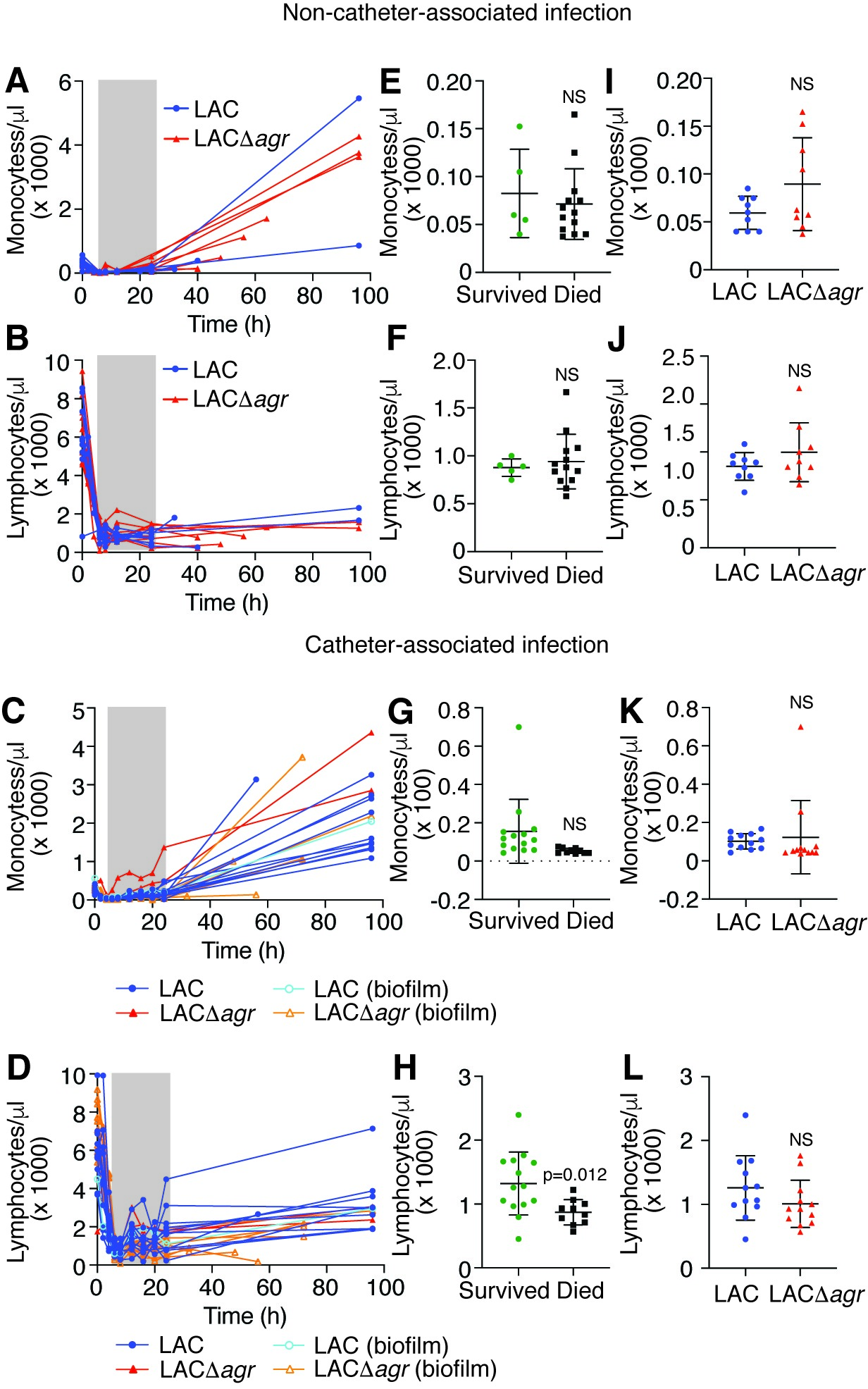

Supplement: S2 Fig — (A-D) Monocyte and lymphocyte numbers over time in the non-catheter-associated (A,B) and catheter-associated (C,D) model. (C,D) Rabbits that developed catheter biofilms are marked by open symbols and light-colored borders. (E-H) Analysis of death versus survival outcome based on average monocyte and lymphocyte numbers for every animal in the time window 6–24 h (grey shading) in panels A-D. (I-L) Analysis of impact of Agr status on average monocyte and lymphocyte numbers for every animal in the time window 6–24 h (grey shading) in panels A through D. (C-L) Statistical analysis is by unpaired two-tailed t-tests. Error bars show the mean ± SD. NS, not significant (p≥0.05). (TIF) [file ppat.1009369.s002.tif]

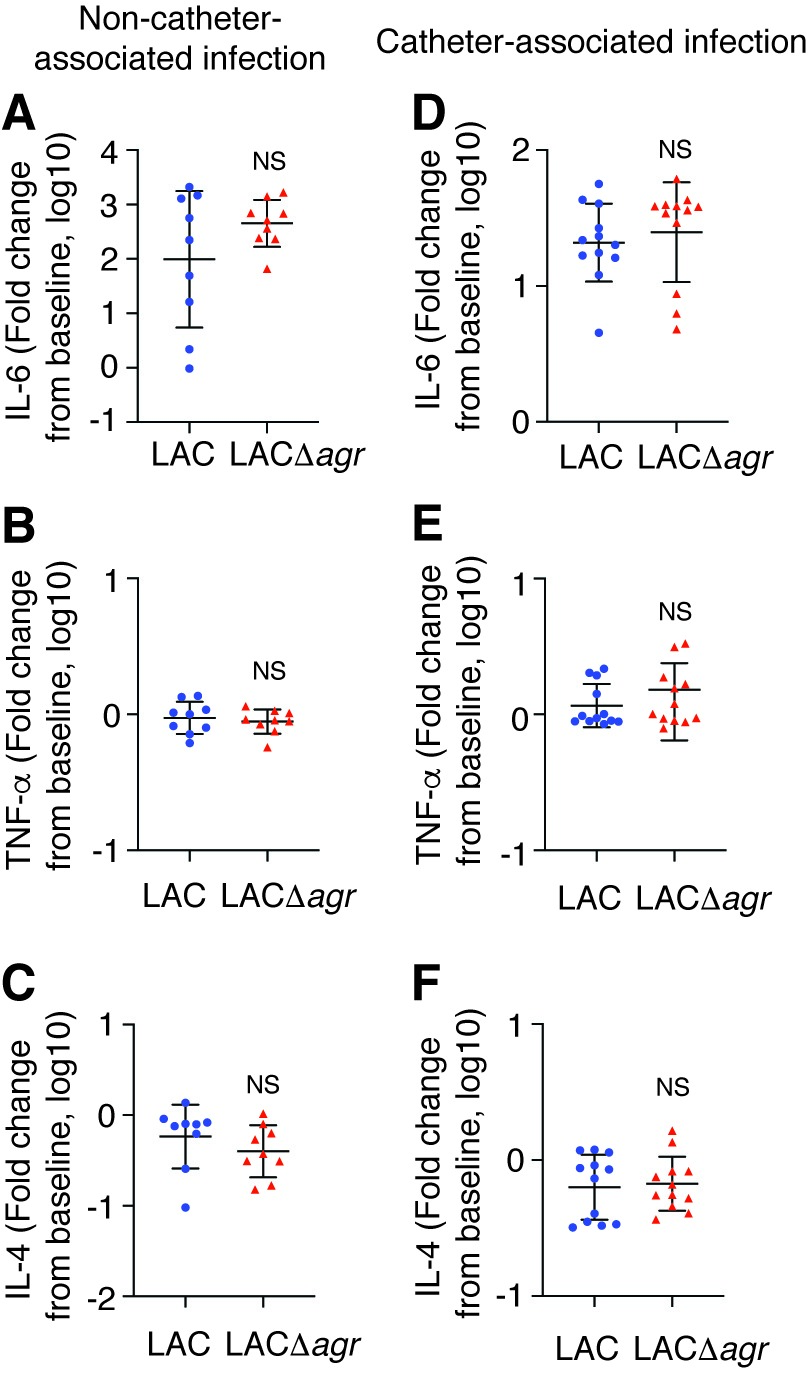

Supplement: S3 Fig — Fold-changes in average cytokine levels for every animal in the rabbit infection models in the early 6–24 h infection window were analyzed dependent on infection group (wild-type versus Δagr-infected animals). Statistical analysis is by unpaired two-tailed t-tests. Error bars show the geometric mean and geometric SD. NS, not significant (p≥0.05). (TIF) [file ppat.1009369.s003.tif]
