# Supplementary material for: Bacterial virulence plays a crucial role in MRSA sepsis
Source: PLoS Pathog. 2021 Feb 25;17(2):e1009369. doi: 10.1371/journal.ppat.1009369 (PMC7942999; doi:10.1371/journal.ppat.1009369)
Supplement: Attachment — Submitted filename: response.docx [file ppat.1009369.s004.docx]

**RESPONSE TO REVIEWERS**

**PLEASE FIND REPLIES BELOW IN BLUE TEXT**

Dear Dr. Otto,

Thank you very much for submitting your manuscript "Bacterial virulence plays a crucial role in MRSA sepsis" for consideration at PLOS Pathogens. As with all papers reviewed by the journal, your manuscript was reviewed by members of the editorial board and by several independent reviewers. In light of the reviews (below this email), we would like to invite the resubmission of a significantly-revised version that takes into account the reviewers' comments.

As you will see from the reviews, all referees thought the work has potentially important implications. However, all three were concerned about the relatively sparse data to support the major conclusions of the paper. In particular, R1 and R2 would like to see more thorough investigation of the catheter biofilms. All reviewers noted that the correlation between leukopenia and mortality was not strongly supported by the data, and R1 points out that the mechanism of leukopenia is not investigated. R3 raises important questions about the robustness of the statistical analysis and the hazards of drawing conclusions on the basis of apparently a single experiment with a small number of animals. Confirming the results of the rabbit experiment in another group of animals would greatly strengthen the conclusions.

Reviewer's Responses to Questions

**Part I - Summary**
Please use this section to discuss strengths/weaknesses of study, novelty/significance, general execution and scholarship.

Reviewer #1: Cheung and colleagues studied staphylococcal pathogenesis in models of catheter and non-catheter related sepsis both in mice and rabbit. To follow virulence functions regulated by agr, particularly toxins, they compared infection induced by WT and agr mutants. Overall, rabbits nicely modeled human sepsis and showed several clinical and lab features that approximated what is seen in human sepsis. For example, like in human infection, low WBC was shown to be a prognosticator of poor outcome, and agr negative S. aureus is associated with Staphylococcal bacteremia. The authors proposed that lysis of WBC by toxins accounted for the low WBC and drives poor outcome, although that needed better support. Finally, because negative agr is associated with worse outcome in catheter infection, the authors caution on the use of quorum sensing blockers for treatment of S. aureus sepsis. Overall, the study has potentially important clinical implications, but mechanistic aspects could be improved.

Reviewer #2: This paper examines the contribution of virulence factors during S. aureus sepsis.
The authors examine the possibility that sepsis is not only the result of excessive inflammation. The authors surmise that bacterial factors also contribute to sepsis. They hypothesize that secreted leukocidins may actively kill leukocytes early during infection exacerbating sepsis. Because of the redundancy of secreted leukocidins, the authors settle into comparing the infectivity of the wild type LAC strain and the isogenic mutant in the global regulator Agr which controls the expression of many leukocidin genes amongst many other genes. In addition, the authors use both a mouse and rabbit models of IV infection with and without catheter induced bacteremia. This is done because mouse leukocytes are in general resistant to S. aureus leukocidins unlike rabbit leukocytes.
The study addresses an important question in the field using a rational design. But, the interpretation of the results is constrained by the small number of data points.

Reviewer #3: Authors present findings of animal models of infection with WT MRSA and an isogenic agr-deletion strain to demonstrate the role of agr-mediated quorum-sensing in the pathogenesis and subsequent outcome of sepsis. Major conclusions are that bacterial leukocidins up-regulated via agr-mediated mechanisms during early sepsis contribute to leuko- and neutropenia that drives fatal outcomes. In addition, a model of IV infection in rabbits with a pre-inserted central venous catheter (CVC) surprisingly shows that agr-deficiency is associated with higher mortality that is associated with biofilm formation. Taken together, the implications of such findings could be highly significant, as they would suggest that treatment modalities targeting S. aureus virulence factors could effectively improve staphylococcal sepsis outcomes, but that targeting quorum-sensing may be the wrong approach in device- or biofilm-associated infection. The experiments were well-executed by researchers with deep experience in the methods and the field, although small numbers of animals, particularly in the catheter-associated infection model, hamper generation of statistically-supported conclusions in several experiments. Such high impact conclusions should be substantiated by more appropriate statistical analyses in some experiments, and this may not be feasible with the numbers of animals used in the experiments represented.

Authors describe two major experiments from which they ultimately draw their conclusions. First, using a mouse model of MRSA sepsis from IV infection with a WT (1 of 10 survived) or an isogenic agr-deletion LAC strain (all survived), they demonstrate the critical role of agr-mediated quorum-sensing in mortality. From blood draws early after infection, they show that survival was associated with higher leukocyte and leukocyte subset numbers. They repeat this experiment with cyclophosphamide administered prior to infection to show that leukocyte-depletion renders infection even with the agr-deletion strain fatal. Taken together, they conclude that leukopenia is associated with fatal outcome in this model, and that agr-regulated expression of leukocidins drives this critical deficit.

In their second experiment, they use a similar infection model with the same MRSA strains in rabbits with and without prior placement of a CVC. In the non-catheter-related infection, mortality was similar but delayed following infection with the agr-deletion strain (3 of 9 survived versus 2 of 9 survivors from WT infection). Surprisingly, in rabbits with a CVC, 6 of 6 animals survived IV infection with the WT strain while only 2 of 6 animals survived IV infection with the agr-deletion strain, and the 4 rabbits who succumbed had biofilm detected in their catheter by CFUs counted from postmortem catheter flushes (while the 2 survivors in this group had no biofilm). Survivors in both the non-catheter- and catheter-related models had higher neutrophil counts than non-survivors. Cytokine evaluation early after infection revealed elevated IL-6 amongst all animals with no difference among survivors and non-survivors but minimal change and no difference in TNFa levels. Taken together, authors conclude that (1) the impact of agr-mediated pathophysiology in non-catheter-related sepsis is weaker in rabbits than in mice and (2) the agr-mediated effect is reversed in catheter-associated sepsis in rabbits and this reversal is due to biofilm formation and (3) early leuko- and neutropenia is associated with mortality in non-catheter and catheter-related infection while levels of canonical inflammatory-associated cytokines were not.

**We like to thank the reviewers and the editor for their positive and encouraging evaluation of our manuscript. We now present a revised version of our manuscript that - as a response to the main criticism pointed out by the editor and several reviewers - includes additional data from 12 rabbits (n=6/group) for the catheter-associated model, taking the total number of rabbits to 12/group for that model. We also added further statistical evaluation as requested. The new statistical evaluation, based on the doubled number of animals, resulted in strong support of our previously stated conclusions.**

**We also changed the manuscript in response to the reviewers’ suggestions as detailed in the following.**

**Furthermore, we would like to apologize for the long turnaround of this revision, which was due to the long time we had to wait until we could start the new rabbit experiments. The NIH for several months only allowed Covid-related animal work to be performed, and even after general approval that non-Covid-related animal work could be resumed there was a long wait period for rabbit experiments due to prioritization of Covid-related work.**

**List of experimental and analysis additions:**

- **Extension of rabbit catheter-related model by 6 rabbits/group. New data are included in Figs. 2C, all panels of Fig. 4, Fig. 5 (panels relating to catheter-associated infection), Fig. 6 (panels relating to catheter-associated infection), Fig. S3, S4, S5 (panels relating to catheter-associated infection)**
- **Addition of Agr status evaluation for all analyses of early infection leukocyte and cytokine concentrations. New data are shown in Fig. 5, Fig. S2, Fig. S4, Fig. S5.**
- **Addition of monocyte and lymphocyte analyses in the rabbit models (Fig. S4).**
- **In all statistical evaluations presented in the graphs, p values were rounded to 2 significant digits and “NS” was marked for not significant (p≥0.05).**

Reviewer's Responses to Questions

**Part II – Major Issues: Key Experiments Required for Acceptance**
Please use this section to detail the key new experiments or modifications of existing experiments that should be absolutely required to validate study conclusions.

Generally, there should be no more than 3 such required experiments or major modifications for a "Major Revision" recommendation. If more than 3 experiments are necessary to validate the study conclusions, then you are encouraged to recommend "Reject".


Reviewer #1: 1) Reduced WBC as a prognosticator of poor outcome (a major point of this paper) is ascribed to toxin mediated cytolytic activity, but no supporting data is provided. The authors referred to a SA pneumonia paper where cytolysis is demonstrated. This needs to be better supported in the bloodstream infection model.

The reviewer is absolutely correct in that there is a clear lack of in-vivo data on the association of leukopenia with S. aureus leukocidins, although this is often stated as a phenomenon associated with systemic disease due to those toxins (such as especially for PVL). The present paper is on Agr as a regulator of all those toxins and we believe it is fair to assume that the observed Agr-associated leukopenia is very likely an effect that is due to leukocidins/PSMs. We hope the reviewer agrees that a direct, quite extensive and laborious assessment of leukopenia in systemic infection using several isogenic deletion mutants in those toxin genes is beyond the scope of the present paper, in particular as such experimentation would have to be performed entirely in rabbits. It will certainly be of great interest to address which of those toxins is mainly responsible for the Agr effect in the future. For the present manuscript, we have changed the wording in the corresponding sentence to emphasize the situation of lacking literature addressing this question.

2) agr mutant caused greater mortality than WT S. aureus in the biofilm model. It makes sense that this could be secondary to slow and prolonged release from the catheter. Did the authors culture the catheter directly to demonstrate improved binding to the catheter, rather than indirectly through blood cultures, to correlate with the higher concentration in the blood?

We do not believe that culturing the catheter would yield valuable results, as one could not deduce what the original level of colonization was. We had in the previous version only performed a qualitative assessment of biofilm formation using a CFU threshold, but in the second batch of rabbits now analyzed, we quantified the CFUs on the catheters in detail. The results are shown in Fig. 4C. The difference between WT and agr mutant infected animals was quite striking.

We note that we now also statistically analyzed correlation between Agr status and death as well as between biofilm formation and death. The correlations were highly significant. This is now included in the main text.

Ideally blocking of biofilm related function within agr would lead to similar outcome as delta agr. At least discuss.

The reviewer is absolutely correct - we would expect the same phenotype from a mutant in which all major Agr-controlled contributors are defective. We have shown in several previous publications that PSMs are by far the most important determinants of the Agr biofilm effect in vivo, as a psm total mutant essentially reproduces the phenotype of an agr mutant. We have discussed that role of PSMs in our manuscript, citing the relevant papers.

Reviewer #2: 1.Fig. 1A and 2B: How do the authors account for the difference in mortality associated with the agr mutant between mice and rabbits? The results seem counter-intuitive given the notion that the mouse should be insensitive to leukocidins controlled by Agr (as pointed by the authors).

We completely agree - this is an interesting effect. We did not previously discuss it because the explanation is a bit difficult for a general audience. At first glance, as the reviewer states, this appears counterintuitive. But it is actually to be expected. One has to distinguish between the absolute effect on pathogenesis (higher in rabbits due to leukocidin activity) and the relative Agr effect (higher in mice due to the presumable sole contributors, PSMs, being much more strongly Agr-affected). We have now added a paragraph to explain this in the discussion:

“Our study revealed differences in mouse versus rabbit test animals regarding the relative impact of Agr during non-catheter-associated infection, inasmuch as the relative impact of Agr was stronger in mice. We believe this to be due to the fact that mice are immune to many leukocidins owing to the lack of responsive receptors (47). While rabbits are therefore overall more sensitive to infection by *S. aureus*, the relative impact of Agr – which increases with the relative Agr dependence of toxin expression – is greater in mice, as in that species it is mostly dependent on PSMs, which are under much stricter Agr control than the bicomponent leukocidins (40, 51). As rabbits more closely resemble humans in their responsiveness to leukocidins, this finding indicates that the impact of Agr on systemic infection may be overestimated in the mouse infection model.”

2.l. 171-173 (Fig. 4): the authors state that 4 of 6 rabbits infected with the agr strain developed biofilms on the device and all these animals succumbed to infection. It would have been nice to report CFUs in biofilms or at least images of LAC and agr biofilms. Presumably some of the LAC infected animals also developed biofilm (Fig. 4A). It is hard to see the blue lines in this graph. Does this mean that biofilm in LAC infected animals do not release bacteria in the bloodstream?

We measured biofilm formation in the first batch of rabbits by analyzing CFU on the catheters, but only determined whether they met our threshold criteria. We now report exact CFU for the second batch of rabbits (see Fig. 4C). We note that the results with the second batch were even clearer (all agr rabbits developed biofilm, no WT rabbits did) and report a statistical analysis of correlation between biofilm formation and death (and between Agr status and death) for all rabbits (n=12/group) in the text.

There was only one rabbit infected with wild-type bacteria (from the first batch) that developed a biofilm according to our threshold criteria. This rabbit, as the reviewer mentions, had only marginal CFU in organs. We deemed it not appropriate to speculate on results obtained in one rabbit only. We do not believe that a wild-type biofilm releases less bacteria; this would be opposite to the results we have shown in previous papers on S. aureus and S. epidermidis (Wang et al. JCI 2011 and Periasamy et al PNAS 2012). The biofilm in that rabbit may have been less pronounced, but we do not have these detailed data. It may also be possible that spontaneous development of quorum cheaters happened in that rabbit, which is a clinically and experimentally reported phenomenon (see for example our paper He et al. Nat Microbiol 2019).

3.Fig. 5: The authors conclude that neutropenia correlates with fatality in both device- and non-device associated sepsis (lines 186-187). However, when looking at Fig. 5D and Fig. 5H, it is hard to make such a conclusion. In panel 5D, animals survived with an average neutrophil count of 2000 per microliter. But in panel 5H, it seems that for the same average, all the animals died; rather 5000 neutrophils per microliter were required for survival.

We are confident that with the analyses we performed, we can come to a clear conclusion about the association of low leukocyte count early in infection and ultimate mortality.

The difference that the reviewer points out can be explained by the somewhat different infection kinetics of a catheter- and non-catheter-associated model. Note that our conclusions are based on analyses within a certain model and not directly comparing raw data of the two models.

4.The authors compare blood cell counts between surviving and dead animals throughout the manuscript. Do the authors observed any sort of cell count correlation when comparing the 2 strains used for infection?

Thanks you for this question. The revised manuscript contains the requested analyses in all instances: Fig. 5I-L, Fig. S2, Fig. S4, Fig. S5. The data are entirely supportive of our conclusions.

Reviewer #3: 1.The data authors cite to justify the conclusion that leukopenia/neutropenia early after infection is associated with morbidity/death are not convincing as shown. If interpreting correctly from their methods description, they included repeated measures from the same 5-6 animals across several 2-hour intervals, but grouped all of these results stratified by survivors and non-survivors for comparison. That seems akin to having several technical replicates (repeated measures from the same animal) represent biological replicates for this critical comparison. This analysis should be more accurately represented, perhaps by measuring the fold-change in cell counts during the first 24 hours in each animal, or using an individual and consistent timepoint for each animal (ie- last CBC prior to moribund or last available for all animals) and comparing these between the groups.

We thought long about how to best analyze our data. A comparison of specific time points, or a an analysis of fold-change for a sort of analysis the reviewer mentions, was hardly possible, because we could not always acquire a complete set of data points for all rabbits for technical reasons and because there were variations looking at one time point only that did not allow for a clear fold-change determination. For those reasons, we analyzed a time window, which allowed for averaging out blips and missing time points. We believe this to be an appropriate analysis under the circumstances of – particularly in the case of the catheter-associated infection - a technically highly challenging model.

We also note that with the addition of further rabbits to the study and more statistical evaluation, the obtained data and statistical comparisons are now even stronger in supporting our conclusions.

2.In general, many of the conclusions inferred from the data in regards to recovered CFUs, WBC and neutrophil trends, and cytokine findings are based on trends without applied statistics (some examples are specifically cited as Minor Issues). This likely reflects lack of power to appropriately apply statistical comparisons between groups due to small numbers, but it generally weakens the strength of several of the major conclusions drawn by the authors.

As requested also by the editor, we now added more rabbits to the difficult catheter-associated model (increasing the number of animals from 6 to now 12 per group). The statistics relating to leukocyte counts and survival now are even more robust in supporting our conclusions. Furthermore, we added statistical evaluation (by Fisher’s exact test, in the text) of the correlation between biofilm formation and death as well as Agr status and death, data which again were in full support of out conclusions. Moreover, we added an analysis of Agr status regarding leukocyte count early in infection (Fig. 5I-L, Fig. S2, Fig. S4, Fig. S5), which are all supportive of our conclusions. Finally, some statements that were not in the center of our conclusions and which were not based on statistical analyses and pointed out by the reviewer in the “minor issues” section, were removed from the discussion (see below).

3.The finding that rabbits with a CVC infected with the WT MRSA strain all survived the infection is very surprising and contrasts directly with the findings from the non-catheter-associated infection, yet there is no description from the authors to explain this finding. It is counter to the conclusion that agr-mediated leukocidin expression causes leuko- and neutropenia that leads to mortality and is not well explained by the possibility that bacteria seed the catheter early and are thus sequestered there since the leukocidins would be expected to be secreted. Likewise, explanation of the decreased impact of agr-deficiency on outcome in the non-catheter-associated infection in rabbits compared to mice is lacking.

As for the apparent contradiction of more rabbits infected with the same amount of inoculum surviving in the catheter-related but more dying in the non-catheter-related model, we believe that sequestration, as the reviewer notes, is actually the most likely underlying reason. We now discussed this in the manuscript (results part). We don’t believe that under a scenario of initial colonization of/sequestration by a catheter, Agr would be active and thus leukocidins necessarily assumed to be secreted. Even if, the more localized secretion in that case may have consequences. In any case, our results are clear and we found it too speculative to further speculate on these potentially underlying issues in the manuscript.

As for the different impact of Agr in the two different model animals, which reviewer 2 also notes, we completely agree - this is an interesting effect. We did not previously discuss it because the explanation is a bit difficult for a general audience. At first glance, as the reviewer states, this appears counterintuitive. But it is actually to be expected. One has to distinguish between the absolute effect on pathogenesis (higher in rabbits due to leukocidin activity) and the relative Agr effect (higher in mice due to the presumable sole contributors, PSMs, being much more strongly Agr-affected). We have now added a paragraph to explain this in the discussion:

“Our study revealed differences in mouse versus rabbit test animals regarding the relative impact of Agr during non-catheter-associated infection, inasmuch as the relative impact of Agr was stronger in mice. We believe this to be due to the fact that mice are immune to many leukocidins owing to the lack of responsive receptors (47). While rabbits are therefore overall more sensitive to infection by *S. aureus*, the relative impact of Agr – which increases with the relative Agr dependence of toxin expression – is greater in mice, as in that species it is mostly dependent on PSMs, which are under much stricter Agr control than the bicomponent leukocidins (40, 51). As rabbits more closely resemble humans in their responsiveness to leukocidins, this finding indicates that the impact of Agr on systemic infection may be overestimated in the mouse infection model.”

**Part III – Minor Issues: Editorial and Data Presentation Modifications**
Please use this section for editorial suggestions as well as relatively minor modifications of existing data that would enhance clarity.

Reviewer #1: 3) While cytolysis is a phenomenon that is readily observed in animals particularly when high inoculums of SA is injected, it is unclear how relevant that is with lower inoculum infections such as bloodstream infection (in humans). Toxins have important pro-inflammatory and cytolytic activity at different concentrations. This could be further discussed.

We agree. We have now inserted a part in the discussion emphasizing the issues the reviewer notes:

“Furthermore, we are aware of the fact that these toxins not only have cytolytic, but also pro-inflammatory capacities, often detected at concentrations much lower than those that are cytolytic (57, 58). Moreover, some toxins, such as PSMs and LukAB/GH, are assumed to contribute to leukocyte lysis after phagocytosis rather than by toxin release in to the bloodstream (59, 60). Which specific leucocidin activities contribute to pathogenesis, including during different experimental setups that may differ for example in the initial bacterial inoculum, is not understood.”

4) Line 204 – “Sepsis has not traditionally been thought to be impacted significantly by bacterial
205 virulence” – Not clear if this is a general consensus. Please provide a reference

We reworded that sentence to tone down the statement. The sentence refers to the following two sentences, where references are provided.

5) Figure 3 and 4 - CFUs collected at time of death are not particularly useful. Probably better in supplement.

We thought much about which of the many data we obtained to include in the main manuscript and which in supplemental. We left these data in the main manuscript, as they not only illustrate the CFU at the time of death, but illustrate the time of death and helps comparing CFU in animals that died versus those that survived.

Reviewer #2: 1.Fig. 1B: Data was not collected at time 0 to ascertain the range of WBCs (neutrophils, monocytes, lymphocytes). The authors assume that all animals add the same number of neutrophils at time 0 but is this truly the case?

Due to the nature of the experiment that requires frequent sampling, limited volumes of blood can only be collected from mice during specified periods based on body weight as imposed by the NIH NIAID Animal Care and Use Committee. In this study, we were unable to obtain samples at time 0 because sampling was prioritized for post-infection time points. However, 3 naïve mice were sacrificed and CBCs analyzed. We found only little variation in the number of WBCs prior to infection.

We also note that the focus of the manuscript is rabbits, where the same type of analysis was performed, blood draws are not as limited as in mice, and values at t=0 are included.

2.Fig. 2: Were bacterial inocula prepared similarly for infections shown in panels B and C?

Yes, exactly the same way.

3.The method regarding mouse infection (l. 302-303) should describe the preparation of S. aureus bacteria. Reference 20 describes a paper for S. epidermidis. It is okay to be specific here and confirm that authors performed tail vein injection.

We now specified tail vein injection and replaced the S. epidermidis reference with a paper on S. aureus where we used the same method.

4.l. 163: replace Dagr with “delta”agr.

Thanks, this was corrected.

5.Some of the sentences read awkwardly throughout the text especially in the Abstract and Author Summary.

We checked the abstract and author summary and introduced several changes. We hope it now reads better.

Reviewer #3: 1.In Figure S1, p-values are described in the figure legend and referenced in the text but are not shown in the images.

These comparisons were all not significant, as stated in the text. We now added “NS” for not significant in all figures where this applies.

2.In Figure 3B, could moribund animals be denoted with empty circles as in Fig S1? It would help clarify the statement that “CFU over the time course of infection in the blood and organs reflected the mortality results, inasmuch as they were (i) increased in moribund animals, at least in the blood and some organs” in lines 165-167.

This was changed as requested.

3.The statement that “Leukocyte and neutrophil numbers decreased in the early hours of infection in the non-catheter-related infection model, while they increased again in survivors (Fig. 5A,B,E,F). This was not observed in the catheter-related model” in lines 179-181 is not clearly supported by the data shown.

We admit this difference is difficult to see from the data. We have changed the wording in this case.

4.The statement that “Concentrations of TNF-a followed a similar pattern but with much lower overall changes (Fig. 6B,H).” in lines 192-193 is not corroborated by the data shown, especially in the non-catheter-associated infection.

We agree; we changed this conclusion.

5.The statement that “However, we observed generally increased concentrations of pro- and decreased concentrations of anti-inflammatory cytokines over the course of the infection. Our results indicate that immune suppression early during infection plays a critical role for the development of sepsis, while later inflammatory phenomena appear associated with a moribund stage” in lines 231-235 are not corroborated by the data shown. In particular, the distinction of later inflammatory phenomena being associated with moribund stage are not supported by either the kinetics as shown or the lack of difference detected in survivors versus non-survivors.

We agree. This was deleted.
