# Supplementary material for: Bacterial virulence plays a crucial role in MRSA sepsis
Source: PLoS Pathog. 2021 Feb 25;17(2):e1009369. doi: 10.1371/journal.ppat.1009369 (PMC7942999; doi:10.1371/journal.ppat.1009369)
Supplement: Attachment — Submitted filename: Reviewers2.docx [file ppat.1009369.s005.docx]

**Part I - Summary**
Please use this section to discuss strengths/weaknesses of study, novelty/significance, general execution and scholarship.

Reviewer #1: The manuscript presents conceptual important findings.

Although the role of toxins has not been fully corroborated, the authors have satisfactorily addressed my concerns.

*Reply: We share the reviewer’s interest in the specific role of the Agr-controlled toxins, which we hope to address in the future.*

Reviewer #3: The authors’ revision addresses many of the questions and concerns raised in review of the original manuscript. Namely, the increased number of animals in the critical catheter-related infection model helps to strengthen many of the conclusions drawn about the differential role of agr-mediated effect on virulence in this model. The additional statistical analyses facilitated by these numbers (such as the correlations between agr status or biofilm formation and mortality) also bolster the strength of these conclusions. And the enhanced description of possible reasons for the differences in the role of agr in mice versus rabbits and in catheter- versus non-catheter-related infections substantiate these findings and provide a clearer understanding of what knowledge gaps remain to more fully understand the mechanisms of pathogenicity mediated by agr-regulated toxins.

*Reply: We understand the reviewer’s concerns. We consulted an NIAID statistician and changed the analysis, as detailed below.*

**Part II – Major Issues: Key Experiments Required for Acceptance**
Please use this section to detail the key new experiments or modifications of existing experiments that should be absolutelyrequired to validate study conclusions.

Generally, there should be no more than 3 such required experiments or major modifications for a "Major Revision" recommendation. If more than 3 experiments are necessary to validate the study conclusions, then you are encouraged to recommend "Reject".

Reviewer #1: (No Response)

Reviewer #3: 1. In reference to the following comment from the original review: “The data authors cite to justify the conclusion that leukopenia/neutropenia early after infection is associated with morbidity/death are not convincing as shown. If interpreting correctly from their methods description, they included repeated measures from the same 5-6 animals across several 2-hour intervals, but grouped all of these results stratified by survivors and non-survivors for comparison. That seems akin to having several technical replicates (repeated measures from the same animal) represent biological replicates for this critical comparison. This analysis should be more accurately represented, perhaps by measuring the fold-change in cell counts during the first 24 hours in each animal, or using an individual and consistent timepoint for each animal (ie- last CBC prior to moribund or last available for all animals) and comparing these between the groups.” Authors’ cite the many challenges in comparing a similar timepoint or a standardized fold-change given difficulty in acquiring a complete set of data due to blood draw limits and technical feasibility in the rabbit experiments. While these challenges can certainly be appreciated, the chosen representation combining multiple timepoints from the same animal in the comparator groups (survived versus died or WT versus delta-agr) may be misleading. Since these data are cited to support one of the major findings from this work, that leukopenia early after infection predicts mortality from S. aureus sepsis, this analysis method should be reconsidered or should perhaps be cited as one possible limitation of the findings as presented.

*Reply:*

*We understand the reviewer’s concern about inflation of data points per animal that enters the statistical analysis, and also the possible over-representation of specific animals in which more time point were taken in the analyzed time window (although missing data points were very rare). On the other hand, we are afraid the sorts of analyses the reviewer suggests all rely on focusing on specific data points, disregarding the problem of natural variation of values and the power that repeated measurement of parameters during the time window in question represents.*

*We consulted an NIAID statistician, who suggested to use the average for the data points within the analyzed time window for every animal rather than including all those time points in the analysis. This circumvents the problems of using any sort of analysis that is dependent on only one time point, while not inflating data points, as only one value for every animal now enters the analysis. We are quite happy with this new sort of analysis, which we believe well addresses the concerns of the reviewer.*

*Naturally, having only one data point per animal reduced the degree of statistical significance of the related comparisons. However, in all rabbit experiments the main comparisons (neutrophils, WBCs, death and agr status analyses) that were significant with the old analysis are still significant. Comparisons for cytokines now are all not significant, in strong support of our conclusions. As for the mouse experiments, we have deleted all similar analyses and only left the survival data with/without cyclophosphamide in the manuscript. This is because the mouse experiments were only performed with n=5/group and comparisons did not have the statistical power required for these analyses. Because our paper focuses on rabbits and the mouse data are merely introductory, but not necessary of our conclusions (except for survival and CY data), we hope this is agreeable.*

*Altogether, the changes related to the new statistical analysis led to a more comprehensive and streamlined manuscript. We particularly note the clear difference between statistically significant association of neutrophil numbers but not cytokine concentrations in the early time window with ultimate death, which the new analysis made even more clear-cut.*

**Part III – Minor Issues: Editorial and Data Presentation Modifications**
Please use this section for editorial suggestions as well as relatively minor modifications of existing data that would enhance clarity.

Reviewer #1: (No Response)

Reviewer #3: 1. The light blue line and open triangle used to denote LAC (biofilm) is too light. This symbol seems to only account for one animal, but it is hard to see in any of the panels.

*Reply: We tried the next, less light blue, but when looking at the outcome, it was more difficult to distinguish from the other blue tone. The light blue we used shows up quite well when printed or in the original tiff files. We left it for now and believe it is best to leave this decision to the production editor.*

2. It seems in Figure 3, the intent is to denote animals that survived with closed symbols and those that did not with open, but this should be clarified either on the graphs or in the legend for Figure 3.

*Reply: Thank you. This was an oversight. It was now included in the legend.*
